# Supplementary material for: Association between a Marine Healing Program and Metabolic Syndrome Components and Mental Health Indicators
Source: Medicina (Kaunas). 2023 Jul 6;59(7):1263. doi: 10.3390/medicina59071263 (PMC10384087; doi:10.3390/medicina59071263)
Supplement: Supplementary file 1 [file medicina-59-01263-s001.zip › medicina-2455660-supplementary.pdf]

Supplementary Table S1. Test of Normality

Variable	Group	Shapiro-Wilk		
		Statistic	df	p-value
Height	Experimental	0.856	10	0.069
	Control	0.640	11	0.000***
Weight	Experimental	0.937	10	0.520
	Control	0.951	11	0.658
WC	Experimental	0.926	10	0.414
	Control	0.667	11	0.000***
SMM	Experimental	0.639	10	0.000***
	Control	0.902	11	0.195
BFM	Experimental	0.593	10	0.000***
	Control	0.885	11	0.120
BMI	Experimental	0.434	10	0.000***
	Control	0.784	11	0.006**
PBF	Experimental	0.517	10	0.000***
	Control	0.863	11	0.063
SBP	Experimental	0.887	10	0.157
	Control	0.638	11	0.000***
DBP	Experimental	0.912	10	0.291
	Control	0.757	11	0.003**
FBG	Experimental	0.951	10	0.675
	Control	0.670	11	0.000***
HbA1c	Experimental	0.887	10	0.157
	Control	0.966	11	0.846
AST	Experimental	0.938	10	0.532
	Control	0.886	11	0.124
ALT	Experimental	0.820	10	0.025*
	Control	0.978	11	0.956
Total Cholesterol	Experimental	0.962	10	0.803
	Control	0.872	11	0.083
TG	Experimental	0.946	10	0.622
	Control	0.833	11	0.026*
HDL-C	Experimental	0.892	10	0.178
	Control	0.903	11	0.199
LDL-C	Experimental	0.915	10	0.321
	Control	0.840	11	0.031*
GGT	Experimental	0.805	10	0.017*
	Control	0.711	11	0.001***
UAC	Experimental	0.966	10	0.856
	Control	0.956	11	0.721
HADS	Experimental	0.936	10	0.514
	Control	0.960	11	0.768
EQ-5D-3L	Experimental	0.906	10	0.256
	Control	0.760	11	0.003**
BEPSI	Experimental	0.840	10	0.044*
	Control	0.834	11	0.026*
CSRS	Experimental	0.844	10	0.049*
	Control	0.907	11	0.227

WC, waist circumference; SMM, skeletal muscle mass; BFM, body fat mass; BMI, body mass index; PBF, percent body fat; SBP, systolic blood pressure; DBP, diastolic blood pressure; FBG, fasting blood glucose; AST, aspartate aminotransaminase; ALT, Alanine aminotransferase; TG, triglyceride; HDL-C, high density lipoprotein cholesterol; LDL-C, low density lipoprotein cholesterol; GGT, gamma-glutamyl transpeptidase; UAC, uric-acid; HADS, hospital anxiety and depression scale; BEPSI, brief encounter psychological instrument; CSRS, cognitive stress response scale; *<0.05, **<0.01, ***<0.001

Supplementary Table S2. Comparison of Pre- and Post-test Results in the Experimental Group: Non-normal Variables

Variable		N	Average	SD	z	p-value
SMM (kg)	pre	10	24.61	5.24	-1.78[b]	0.075
	pos	10	21.53	8.09		
BFM (kg)	pre	10	20.41	7.10	-1.72[b]	0.085
	pos	10	18.03	8.88		
BMI (kg/m^2)	pre	10	25.56	2.68	-2.09[b]	0.036*
	pos	10	22.63	8.31		
PBF (%)	pre	10	31.81	9.20	-1.17[b]	0.236
	pos	10	27.89	12.72		
AST (IU/L)	pre	10	20.50	9.10	-1.56[b]	0.120
	pos	10	18.40	5.36		
GGT (IU/L)	pre	10	20.20	7.97	-2.52[b]	0.012*
	pos	10	16.30	4.22		
BEPSI (score)	pre	10	9.40	2.63	-2.55[b]	0.011*
	pos	10	7.10	1.85		
CSRS (score)	pre	10	10.60	13.01	-.893[b]	0.372
	pos	10	8.70	10.07		

SMM, skeletal muscle mass; BFM, body fat mass; BMI, body mass index; PBF, percent body fat; AST, aspartate aminotransaminase; GGT, gamma-glutamyl transpeptidase; BEPSI, brief encounter psychological instrument; CSRS, cognitive stress response scale; *<0.05 ; b: Based on negative ranks

Supplementary Table S3. Comparison of Pre- and Post-test Results in the Control Group: Non-normal Variables

		Average	SD	z	p-value
Height (cm)	pre	153.46	7.31	-1.753^{b}	0.080
	pos	154.75	7.68		
WC (cm)	pre	94.96	7.08	$-.222^{c}$	0.824
	pos	95.09	6.37		
BMI (kg/m^2)	pre	27.45	4.03	-1.382^{c}	0.167
	pos	26.67	3.88		
SBP (mmHg)	pre	148.27	22.86	-2.397^{c}	0.017*
	pos	122.91	44.40		
DBP (mmHg)	pre	82.27	19.95	-1.159^{c}	0.247
	pos	71.45	25.59		
FBG (mg/dL)	pre	107.91	12.97	$-.533^{b}$	0.594
	pos	103.73	41.00		
TG (mg/dL)	pre	103.73	33.43	-1.646^{b}	0.100
	pos	131.18	52.15		
LDL-C (mg/dL)	pre	96.73	34.68	-2.449^{c}	0.014*
	pos	84.18	29.94		
GGT (IU/L)	pre	24.73	11.42	$-.060^{c}$	0.952
	pos	26.36	16.84		
EQ-5D-3L (score)	pre	7.09	1.81	-2.047^{b}	0.041*
	pos	5.45	2.21		
CSRS (score)	pre	8.91	8.96	$-.534^{c}$	0.594
	pos	9.27	9.47		

BMI, body mass index; SBP, systolic blood pressure; DBP, diastolic blood pressure; FBG, fasting blood glucose; TG, triglyceride; LDL-C, low density lipoprotein cholesterol; GGT, gamma-glutamyl transpeptidase; CSRS, cognitive stress response scale; *<0.05 ; b: Based on negative ranks ; c: Based on positive ranks

Supplementary Table S4. Comparison of Pre- and Post-test Results Between the Experimental and Control Groups: Non-normal Variables

Variable	Group	pre test		post test		Mean Rank	Z	p-value
		Mean	SD	Mean	SD			
Height (cm)	Exp.	157.87	7.07	158.18	6.453	11.65	-0.507	0.612
	Cont.	153.46	7.31	154.75	7.675	10.41		
WC (cm)	Exp.	92.25	6.11	90.90	4.514	9.55	-1.022	0.307
	Cont.	94.96	7.08	95.09	6.371	12.32		
SMM (kg)	Exp.	24.61	5.24	21.53	8.085	14.15	-2.220	0.026*
	Cont.	21.33	3.21	22.13	3.063	8.14		
BFM (kg)	Exp.	20.41	7.10	18.03	8.88	11.65	-0.458	0.647
	Cont.	25.06	8.39	23.97	8.08	10.41		
BMI (kg/m^2)	Exp.	25.56	2.68	22.63	8.31	12.4	-0.988	0.323
	Cont.	27.45	4.03	26.67	3.88	9.73		
PBF (%)	Exp.	31.81	9.20	27.89	12.72	11.1	-0.07	0.944
	Cont.	38.24	8.85	36.38	8.51	10.91		
SBP (mmHg)	Exp.	152.70	22.34	134.20	9.05	11.55	-0.388	0.698
	Cont.	148.27	22.86	122.91	44.40	10.5		
DBP (mmHg)	Exp.	81.60	15.95	78.10	10.53	10.55	-0.317	0.751
	Cont.	82.27	19.95	71.45	25.59	11.41		
FBG (mg/dL)	Exp.	121.50	23.80	117.80	19.64	12.1	-0.778	0.437
	Cont.	107.91	12.97	103.73	41.00	10		
ALT (IU/L)	Exp.	20.50	9.10	18.40	5.36	13.55	-1.808	0.071
	Cont.	26.91	10.23	28.00	11.53	8.68		
TG (mg/dL)	Exp.	113.10	57.22	79.50	40.32	14.7	-2.606	0.009**
	Cont.	103.73	33.43	131.18	52.15	7.64		
LDL-C (mg/dL)	Exp.	101.80	39.63	101.90	39.89	9	-1.411	0.158
	Cont.	96.73	34.68	84.18	29.94	12.82		
GGT (IU/L)	Exp.	20.20	7.97	16.30	4.22	14.05	-2.167	0.03*
	Cont.	24.73	11.42	26.36	16.84	8.23		
EQ-5D-3L (score)	Exp.	7.30	1.34	5.90	1.37	11.3	-0.216	0.829
	Cont.	7.09	1.81	5.45	2.21	10.73		
BEPSI (score)	Exp.	9.40	2.63	7.10	1.85	11.7	-0.501	0.617
	Cont.	10.27	3.50	8.00	2.24	10.36		
HADS (score)	Exp.	10.80	7.33	7.30	6.31	12.95	-1.38	0.167
	Cont.	12.27	6.92	7.82	5.74	9.23		

WC, waist circumference; SMM, skeletal muscle mass; BFM, body fat mass; BMI, body mass index; PBF, percent body fat; SBP, systolic blood pressure; DBP, diastolic blood pressure; FBG, fasting blood glucose; ALT, Alanine aminotransferase; TG, triglyceride LDL-C, low density lipoprotein cholesterol; GGT, gamma-glutamyl transpeptidase; BEPSI, brief encounter psychological instrument; HADS, hospital anxiety and depression scale, HADS; *<0.05, **<0.01.
